# Editorial: Tremor Syndromes: Current Concepts and Future Perspectives

**DOI:** 10.3389/fneur.2021.752463

**Published:** 2021-09-27

**Authors:** Sanjay Pandey, Julián Benito-León, Sheng-Han Kuo

**Affiliations:** ^1^Department of Neurology, Govind Ballabh Pant Postgraduate Institute of Medical Education and Research, New Delhi, India; ^2^Department of Neurology, University Hospital, Madrid, Spain; ^3^Center for Networked Biomedical Research on Neurodegenerative Diseases (CIBERNED), Madrid, Spain; ^4^Department of Medicine, Complutense University, Madrid, Spain; ^5^Department of Neurology, Columbia University, New York, NY, United States; ^6^Initiative for Columbia Ataxia and Tremor, Columbia University, New York, NY, United States

**Keywords:** tremor, neurophysiology, essential tremor, neuroimaging, genetics

A tremor is a rhythmic, oscillatory movement of a body part produced by alternating or synchronous contractions of antagonist muscles (1). It is the most common movement disorder and can be classified according to its phenomenology, distribution, frequency, or etiology. Phenomenologically there are two major categories of tremors: rest tremors and action tremors. Action tremors can be subdivided into postural, kinetic, isometric, and task- or position-specific tremors. In the last decade, there have been many advancements in the field of tremors including neurophysiology, neuroimaging, and genetics (2). The Frontiers Research Topic “*Tremor Syndromes: Current Concepts and Future Perspectives”* has been published to highlight the current knowledge and literature in the field of tremor research. We have been fortunate that some of the leading researchers and working groups have made outstanding contributions. In this regard, open access publication has clear advantages to spread the knowledge and update the field on the recent advances. This special issue has systematic reviews and original articles covering a wide range of subjects related to tremor research. In a review article, Lenka and Jankovic have discussed different types of tremor syndromes including the recent tremor classification. The first attempt to classify tremors was done in 1998 when consensus criteria were published by the Movement Disorders Society (3). This classification was based on the distinction between rest, postural, kinetic, and intention tremors with additional data from medical history and neurologic examination. However, subsequent advances highlighted the limitations of these criteria. To overcome these, a new consensus criterion for classifying tremors were published recently (2018), and it was based on axis I (clinical characteristics, including historical features, tremor characteristics, associated signs, and laboratory tests) and axis II [etiology (acquired, genetic, or idiopathic)] (4). This tremor classification has many new additions, including a syndrome-based approach, an updated definition of “Essential tremor,” a new terminology “Essential tremor plus (ET plus),” and a new category “Indeterminate tremor.” The new classification is certainly an important advancement adding more clarification and a clinic-based approach. However, there are some controversies mainly focusing on the new terminology “Essential tremor plus” (5). The definition of ET plus is based on the identification of soft signs including questionable dystonia. In another review article Louis, the author has provided excellent evidence based on clinical, etiological, and pathophysiological studies explaining the heterogeneity across ET patients. In the past decade, there has been great advancement in the understanding of the pathophysiology of ET. Buijink et al. have published an explorative study hypothesizing that inhibitory gamma-aminobutyric acid (GABA) and excitatory Glx (glutamate + glutamine) levels in the dentate nuclei of the cerebellum could be differentially altered in ET patients responsive to either β-adrenergic blockers or anticonvulsants. They compared ET patients using either propranolol, or anticonvulsants and healthy controls by measuring GABA, glutamate, and N-acetyl-L-aspartate (NAA) levels in the deep cerebellar nuclei using ^1^H-magnetic resonance spectroscopy and observed no group differences and no correlation with tremor severity. These data could provide imaging evidence of the heterogeneity of ET. In a systematic review, Holtbernd and Shah have summarized structural, functional, and metabolic neuroimaging studies. They have concluded that there is robust evidence indicating that the cerebellum plays a key role within a multiple tremor oscillator network in ET. However, the dopaminergic and iron imaging do not suggest any substantial overlap of ET and PD pathophysiology. In another study, Becktepe et al. have found evidence for a direct association between white matter hyperintensities volume and tremor severity in an MRI study on 47 elderly ET patients. Lesions in the Guillain- Mollaret triangle frequently cause various types of tremors, but their pathophysiology is poorly understood. In a systematic review, Kakei et al. have proposed that tremor results from errors in predictions carried out by the cerebellar circuitry. Deep brain stimulation (DBS) of the ventralis intermedius (VIM) nucleus of the thalamus and the posterior subthalamic area (PSA) is effective in ET treatment (6). In a research article, Kim et al. have compared the stimulation-induced side effects of DBS targeting VIM and PSA areas. They hypothesized that changing active DBS contacts to simultaneous targeting of VIM and PSA may help ameliorate stimulation-induced side-effects. In another review article, Peters and Tisch have summarized the prevalence, risk factors, and long-term outcomes of habituation after DBS in tremor syndromes. The authors have provided some evidence that dystonic tremor and ET may be more susceptible to habituation than Parkinsonian tremor. Transcranial magnetic stimulation (TMS) is a non-invasive brain stimulation technique that has been used for a better understanding of tremor pathophysiology. In a review article, Frey et al. have provided some evidence that repetitive TMS (rTMS) pulses can modulate brain functions through plasticity effects and may provide some therapeutic benefits. Wearable devices have been used for the assessment of tremors. In a review article, Vescio et al. have highlighted the use of wearable technologies for differential diagnosis of tremors. They have also considered possible future use based on inertial sensing for measuring tremors. In another review article, Lorra-Millan et al. have demonstrated the feasibility of managing upper limb tremors through wearable technologies that suppress tremors by modifying limb biomechanics.

Several important themes have emerged from these important research papers and review articles. First, we have a better understanding of the tremor phenomenology and phenotypes. Second, there is growing evidence of the involvement of newer networks in the pathogenesis of tremors. Also, the neuropathologic changes observed in ET patients have helped us to identify pathologic endophenotypes that may allow for the recognition of distinct genetic or clinical variants. Third, interest has grown in the use of novel technologies in tremor treatment and finding new targets and treatment strategies. These findings will be helpful in collaborative and coordinated research on a multinational level. That will also help in standard data collection using common data elements for clinical, neurophysiological, genetic, and pathological studies. Future prospective studies recruiting a large cohort of patients may be planned to collect bio-samples, characterize the natural history of tremor syndrome, identify the pathophysiological mechanism and investigate potential etiologies of various phenotypes (2, 5).

Finally, an inclusive acknowledgment is due to the authors and reviewers who have contributed to this Research Topic. Their honest efforts and commitment have been truly admirable.

## Author Contributions

SP, JB-L, and S-HK have contributed to manuscript writing, editing, and critique. All authors contributed to the article and approved the submitted version.

## Conflict of Interest

The authors declare that the research was conducted in the absence of any commercial or financial relationships that could be construed as a potential conflict of interest.

## Publisher's Note

All claims expressed in this article are solely those of the authors and do not necessarily represent those of their affiliated organizations, or those of the publisher, the editors and the reviewers. Any product that may be evaluated in this article, or claim that may be made by its manufacturer, is not guaranteed or endorsed by the publisher.
